# F_0_F_1_-ATPase Contributes to the Fluoride Tolerance and Cariogenicity of *Streptococcus mutans*

**DOI:** 10.3389/fmicb.2021.777504

**Published:** 2022-01-31

**Authors:** Cheng Li, Cai Qi, Sirui Yang, Zhengyi Li, Biao Ren, Jiyao Li, Xuedong Zhou, Huawei Cai, Xin Xu, Xian Peng

**Affiliations:** ^1^State Key Laboratory of Oral Diseases and National Clinical Research Center for Oral Diseases, West China Hospital of Stomatology, Sichuan University, Chengdu, China; ^2^Department of Cariology and Endodontics, West China Hospital of Stomatology, Sichuan University, Chengdu, China; ^3^Laboratory of Nuclear Medicine, Department of Clinical Nuclear Medicine, West China Hospital, Sichuan University, Chengdu, China

**Keywords:** *Streptococcus mutans*, fluoride(s), genomics, caries, antimicrobials

## Abstract

The phenotypic traits of *Streptococcus mutans*, such as fluoride tolerance, are usually associated with genotypic alterations. The aim of this study was to identify adaptive mutations of *S. mutans* to gradient fluoride concentrations and possible relationships between the mutations and fluoride tolerance. We identified a highly resistant *S. mutans* strain (FR1000) with a novel single nucleotide polymorphism (SNP, −36G→T) in the promoter region of F_0_F_1_-ATPase gene cluster (*SMU_1527-SMU_1534*) resistant to 1,000 ppm fluoride using the whole-genome Illumina PE250 sequencing. Thus, a −36G→T F_0_F_1_-ATPase promoter mutation from the parental strain *S. mutans* UA159 was constructed and named UA159-T. qRT-PCR showed that the F_0_F_1_-ATPase gene expression of both FR1000 and UA159-T was up-regulated, and fluoride tolerance of UA159-T was significantly improved. Complementation of Dicyclohexylcarbodiimide (DCCD), a specific inhibitor of F_0_F_1_-ATPase, increased fluoride susceptibility of FR1000 and UA159-T. Intracellular fluoride concentrations of fluoride tolerance strains were higher compared to UA159 strain as demonstrated by ^18^F analysis. Further validation with rat caries models showed that UA159-T caused more severe caries lesions under fluoride exposure compared with its parental UA159 strain. Overall, the identified −36G→T mutation in the promoter region of F_0_F_1_-ATPase gene drastically contributed to the fluoride tolerance and enhanced cariogenicity of *S. mutans*. These findings provided new insights into the mechanism of microbial fluoride tolerance, and suggested F_0_F_1_-ATPase as a potential target for suppressing fluoride resistant strains.

## Introduction

Fluoride is a ubiquitous compound in the environment, which is found in soil, water, and air (Davison and Weinstein, 2004; Jagtap et al., 2012). It is also widely used in many oral hygiene products, such as toothpaste, mouthwash, and gel (Anusavice et al., 2016). *Streptococcus mutans* is the principal causative agent of dental caries in humans (Hudson and Curtiss, 1990) and fluoride is the most widely used chemical agent for controlling the disease (Chansley and Kral, 1989). It is because that fluoride can inhibit oral bacterial growth and metabolism, protect dental enamel from demineralization and enhance the remineralization process (Buzalaf et al., 2011). The antimicrobial action of fluoride is pH dependent (Sutton et al., 1987). The *pKa* of fluoride is 3.15, and fluoride cannot permeate bacterial cell membranes at pH 7.0. Gutknecht and Walter (1981) reported that the permeability coefficient of synthetic membranes for hydrogen fluoride (HF) was about 1 × 10^7^ times that of fluoride. HF is formed when the oral pH drops as a result of carbohydrate fermentation by *S. mutans*. Then, HF easily crosses the cell membrane and enters the bacterium (Loesche, 1986). Inside the cell, HF dissociates, forming a proton (H^+^) and a fluoride ion (F^–^) in the relatively alkaline cytoplasm (Hamilton, 1990). F^–^ acts as an enzyme inhibitor while H^+^ acidifies the cytoplasm, and they both reduce protons extruded through the cell membrane by inhibiting F_0_F_1_-ATPase, which is a membrane channel (Liao et al., 2017). Enolase, an enzyme involved in glycolysis pathway and glucose uptake, is competitively inhibited by F^–^ (Guha-Chowdhury et al., 1997) and indirectly inhibited by low cytoplasm pH resulting from H^+^ accumulation (Belli et al., 1995; Marquis et al., 2003). Inhibition of this enzyme results in a reduction of sugar uptake and glycolysis (Sutton et al., 1987). Moreover, fluoride modulates metabolism by binding to pyrophosphatase in the presence of Mn^2+^ (Marquis et al., 2003). Also, fluoride inhibits alkali production through the inhibition of urease and the arginine deiminase system (ADS), which requires low pH values (Curran et al., 1998; Burne and Marquis, 2000). In summary, the antibacterial effect of fluoride depends on the influx of HF in an acidic environment. The intracellular F^–^ and H^+^ can directly or indirectly affect enzymatic activities and physiological processes, thereby affecting the biological function of *S. mutans.*

Usage of high fluoride concentrations in oral hygiene products results in fluoride-resistant strains, thus effectiveness of these products is greatly comprised. Fluoride resistance can either be classified as transient or permanent. Transient resistance, acquired through phenotypic adaptation, is lost after 1–7 generations in a fluoride-free medium (Streckfuss et al., 1980). Fluoride-resistant strains isolated clinically were likely to have transient fluoride resistance. Transient fluoride-resistant *S. mutans* strains, which can resist to 300–600 ppm fluoride, have been isolated from xerostomia patients (Streckfuss et al., 1980; Brown et al., 1983). Transient fluoride resistance may be the result of environmental adaptation and is related to the horizontal transfer of bacterial plasmids. These strains will lose its plasmid in the absence of fluorine stimulation and revert to fluorine-sensitive strains (Streckfuss et al., 1980). Permanent fluoride resistance results from chromosomal alterations (Mitsuhata et al., 2014; Liao et al., 2015). van Loveren et al. (1991) defined the genotypic fluoride resistance as a stable resistance that can persist for at least 20 generations in a fluoride-free medium. Most laboratory-derived fluoride-resistant strains showed stable fluoride resistance, and were used to study cariogenicity, strain fitness, and mechanisms of acquired fluoride resistance. *In vitro* induction and further artificial selection were used to isolate stable fluoride-resistant strains. van Loveren et al. (1991) directly transferred fluorine-sensitive parent strains *S. mutans* C180-2 from fluoride-free medium to 1,000 ppm fluoride medium to obtain fluoride-resistant strains. Brussock and Kral (1987), Zhu et al. (2012), Liao et al. (2015), and Mieher et al. (2018) cultivated fluorine-sensitive parent strains GS-5, C180-2, Ingbritt, and UA159 on medium containing increasing concentrations of NaF (0−1,000 ppm, in increments of 50 ppm), and then isolated fluoride-resistant strains on plate. Until now, researchers have found SNPs in several genes from isolated fluoride-resistant strains, including *eno* and *pykF* (encoding two glycolytic enzymes, namely enolase and pyruvate kinase), *glpF* (encoding a glycerol uptake facilitator protein), and *eriC^F^* (encoding fluoride antiporters, EriC*^F^*) (Liao et al., 2015, 2018). Only *eriC^F^* has been clearly confirmed to be linked to fluoride resistance (Liao et al., 2016; Men et al., 2016; Murata and Hanada, 2016). Mechanisms of fluoride response by cells are still not clear.

F_0_F_1_-ATPase, a membrane-bound proton translocating ATPase, is a well-known aciduric virulence factor of *S. mutans* (Bender et al., 1986). Previous studies showed that it also has a potential regulatory effect on fluoride resistance. In the fluoride environment, the influx of HF, as well as the inhibition of the proton-extruding F_0_F_1_-ATPase, leads to the acidification of the cytoplasm and the reduction in acidurance of *S. mutans* (Bender et al., 1986; Marquis, 1990; Van Loveren, 2001). However, F_0_F_1_-ATPase in a fluoride-resistant strain was found to be insensitive to fluoride at pH 5.0 while the corresponding wild-type strain was sensitive under the same pH condition (Hoelscher and Hudson, 1996). Previous studies on fluoride inhibition of F_0_F_1_-ATPase were done either with the purified enzyme or in permeabilized cells (Sutton et al., 1987; Marquis, 1995; Pandit et al., 2013). The inhibition of F_0_F_1_-ATPase in intact cells remains unknown. Vanloveren et al. (2010) speculated that fluoride-resistant strains may have a both fluorine-resistant and acid-resistant F_0_F_1_-ATPase transport system. On the other hand, F^–^ can bind to F_0_F_1_-ATPase as AlF^–^, and replaces PO_4_^3–^, which binds to ADP at the active site. Thus, it can inhibit F_0_F_1_-ATPase by forming an inactive complex. So, Hoelscher and Hudson (1996) speculated that the reason why F_0_F_1_-ATPase in fluoride-resistant strains is insensitive was that their structure of F_0_F_1_-ATPase has changed, and fluoride was unable to bind to the active site. Moreover, Miwa et al. (1997) speculated that expression of F_0_F_1_-ATPase was upregulated in fluoride-resistant strains, which increased the number of the enzyme to antagonize the inhibitory effect of fluoride. However, in previous studies on fluoride-resistant strains, no mutation was found in the *atpHGFEDCBA* operon.

In this study, we found a novel adaptive F_0_F_1_-ATPases point mutation, which significantly altered fluoride tolerance and cariogenicity. This article provided new insights on mechanisms of microbial fluoride resistance.

## Materials and Methods

### Bacterial Strains and Growth Conditions

*S. mutans* UA159 strains were provided by State Key Laboratory of Oral Diseases of Sichuan University. Bacteria were grown routinely in Tryptone soya broth (TSB, OXOID, Basingstoke, England) containing 0/300/600/1,000 ppm Sodium Fluoride (NaF, KESHI, Chengdu, China) and in an anaerobic chamber (10% H_2_, 5% CO_2_, and 85% N_2_; Thermo Fisher Scientific, Inc., Waltham, MA, United States) at 37°C. Tryptone soya agar (TSA, OXOID, Basingstoke, England) containing 300/600/1,000 ppm NaF were used for bacterial plating and isolation. To be specific, first of all, the wild-type strain UA159 was cultivated in fluoride-free TSB in a 15 mL centrifuge tube (NEST, Jiangsu, China) in an anaerobic chamber at 37°C for 16 h. Then, 1 mL bacterial suspension was added to 9 mL TSB (1:10 dilution) and cultivated at the same conditions for 2–3 h until exponential phase (*OD*_600_ = 0.6). Next, 1 mL exponential-phase UA159 was added and cultivated in 9 mL TSB containing 300 ppm NaF for 16 h until OD_600_ reached 0.6. Then 200 μL bacterial suspension was plated in TSA containing 300 ppm NaF in an anaerobic chamber at 37°C for 48 h. Thus, transient fluoride-resistant strains which can tolerate 300 ppm NaF was isolated. To get stable fluoride-resistant strains, isolates were cultivated in 10 mL fluoride-free TSB in a 15 mL centrifuge tube for 16 h at the same conditions for 20 passages, respectively. At last, stable 300 ppm-fluoride-resistant strains were isolated in TSA plates containing 300 ppm NaF and named FR300. A few (10–20) isolates of FR300 were obtained and one was picked for whole-genome sequencing. Following the same procedure, FR300 was cultivated in TSB containing 600 ppm NaF for 16 h, and then plated in TSA containing 600 ppm NaF. Transient fluoride-resistant strains were cultivated in fluoride-free TSB for 20 passages, and then plated in TSA containing 600 ppm NaF to get stable 600 ppm-fluoride-resistant strains, namely FR600. Similarly, 1,000 ppm-fluoride-resistant strains were derived from FR600 and named FR1000. One isolate of FR600 and FR1000 each was picked for whole-genome sequencing (Supplementary Figure 1 showed the isolation procedure from gradient fluoride concentrations). Growth conditions (gradient fluoride concentrations of the medium, the anaerobic environment and the temperature) were carefully controlled in all phenotypic experiments. Bacterial strains utilized in this study are listed in Supplementary Table 3.

### Whole-Genome Sequencing

Genomic DNA was extracted using Wizard^®^ Genomic DNA Purification Kit (Promega) according to the manufacturer’s protocol. Purified genomic DNA was quantified with TBS-380 fluorometer (Turner BioSystems Inc., Sunnyvale, CA). High quality DNA (OD260/280 = 1.8–2.0, >1μg) was considered suitable for further experiments.

For Illumina sequencing, at least 1 μg genomic DNA for each strain was used to construct sequencing library. DNA samples were sheared into 400–500 bp fragments using a Covaris M220 Focused Acoustic Shearer following the manufacture’s protocol. Illumina sequencing libraries were prepared from the sheared fragments using the NEXTflex™ Rapid DNA-Seq Kit. Briefly, 5′ prime ends were first end-repaired and phosphorylated. Next, the 3′ ends were A-tailed and ligated to sequencing adapters. The next step was the enrichment of the adapters-ligated products using PCR. The prepared libraries were used for paired-end Illumina sequencing (2 × 150 bp) on an Illumina HiSeq X Ten machine.

Raw Illumina paired-end reads were demultiplexed using barcodes and quality trimmed using Trimmomatic (Bolger et al., 2014). Trimmed reads were mapped to reference genome of *S. mutans* UA159 with the updated RefSeq annotation using Burrows-Wheeler Aligner (Li and Durbin, 2009). The resulting SAM files were converted to BAM files and indexed using SAMtools (Li et al., 2009). The platform was used to identify GATK SNPs and INDELs (DePristo et al., 2011) with default parameters except that the minimum phred-scaled confidence threshold was 50, which means only the variants with ≥50% frequency were selected. The whole-genome sequencing data of FR300, FR600, and FR1000 is available on the National Center for Biotechnology Information (NCBI) Sequence Read Archive Database, accession number *SRR13846722, SRR13846723, and SRR13846724*.

### Quantitative Real-Time PCR Assays

Standard magnetic bead-based procedures described previously (Osman et al., 2012) were used for tissue homogenization, RNA extraction and quantitative real-time PCR (qRT-PCR). Strains were grown in Brain Heart Infusion (BHI, OXOID, Basingstoke, England) broth until exponential phase (OD_600_ = 0.6). 50 mL culture was centrifuged at 4,000 g for 10 min at 4°C. Pellets were added to fastrep tubes (Betin, Shanghai, China) containing 0.1 mm magnetic beads (Betin, Shanghai, China). Total RNA was extracted by high pressure homogenization using the Genejet RNA kit (Thermo Scientific, MA, United States). Genomic DNA contamination was removed, and cDNA was synthesized using PrimeScript RT reagent Kit with gDNA Eraser (Takara, Shiga, Japan). qRT-PCR analyses were conducted using TB Green Premix Ex TaqII (Tli RNaseH Plus) (Takara, Shiga, Japan). mRNA expression levels of the *atpH* gene were normalized using *gyrA* as an internal control (Min et al., 2009; Hossain and Biswas, 2012). The primers utilized are listed in Supplementary Table 4. The experiment was independently repeated three times with four replicates per group (UA159/FR600/FR1000/UA159-T, *N* = 12 per group). IBM SPSS Statistics 24 was employed for statistical analysis using One-way ANOVA to compared groups. Dunnett’s T3 was used for multiple comparisons after ANOVA (heterogeneity of variance). A *P*-value of 0.05 was considered significant.

### Mutant Strain Construction

We constructed a −36G→T F_0_F_1_-ATPases promoter mutation from *S. mutans* UA159 and named it UA159-T. Specific sequences atpH-pTune were synthesized (TSINGKE, Chengdu, China) and 1 μL of these was added to 300 μL UA159 in early exponential phase (OD_600_ = 0.3) with 1 μL competence stimulating peptide (CSP-18, 1 μg/μL, Peptide Pharmaceutical Technology Co., Ltd, Zhengzhou, China). Then, they were cultivated in a 1.5 mL centrifuge tube (NEST, Jiangsu, China) in an anaerobic chamber at 37^°^C for 2 h. In the two control groups, UA159 was cultivated without atpH-pTune, or without neither atpH-pTune nor CSP-18. Then 200 μL bacterial suspension of the three groups was plated in TSA containing 1,000 ppm NaF, respectively, and cultivated in an anaerobic chamber at 37^°^C for 48 h. Thus, fluoride-resistant colonies were isolated in the experimental group. PCR products (Primer atpH-seqFor and atpH-seqRev) were submitted to a sequencing company (TSINGKE, Chengdu, China) to test the validity of sequences. The experiment was independently repeated three times and three isolates were picked for sequencing each time. Interestingly, all the nine isolates showed one −36G→T promoter point mutation, indicating the point mutation could be critical to fluoride resistance. The primers utilized are listed in Supplementary Table 4.

### Minimum Inhibitory Concentration Assays

MIC assays were performed as previously described (Suntharalingam et al., 2009) with some modifications. Briefly, strains (UA159/FR600/FR1000/UA159-T) were grown in TSB overnight until exponential phase (OD_600_ = 0.6) and then diluted with TSB in the proportion of 1:100. We subsequently prepared a 96-well culture plate (Corning-Costar, NY, United States) containing 50 μL TSB, supplemented with 3/4-fold serial dilutions of NaF. The concentration of NaF was 8,000 ppm in column 1 and 0 ppm in column 12. Then, 50 μL diluted bacterial suspension was added to each well. Thus, the concentration of NaF became 4,000 ppm in column 1 but that in column 12 was still 0 ppm. After incubation at 37°C in an anaerobic chamber for 24 h, bacterial growth was spectrophotometrically measured with a microtiter plate reader at an absorbance of 600 nm. The OD_600_ readings for the wells with medium only were subtracted to account for background signals. The minimum inhibitory concentration (MIC) value was determined as the concentration that reduces 90% OD_600_ value of the fluoride-free control. The experiment was independently repeated three times with four replicates per group (*N* = 12 per group and per fluoride concentration).

### Fluoride Inhibitory Assays

Fluoride inhibitory assays were designed based on MIC tests with Dicyclohexylcarbodiimide (DCCD, Solarbio, Beijing, China), a specific F_0_F_1_-ATPase inhibitor. Strains (UA159/UA159-T) were grown in TSB until exponential phase (OD_600_ = 0.6) and then were diluted with TSB at a ratio of 1:100. A 96-well culture plate (Corning-Costar, NY, United States) containing 50 μL TSB, supplemented with DCCD and 1/2-fold serial dilutions of NaF was prepared. Fifty microliter diluted bacterial suspension was added to each well to a final concentration of 0, 125, 250 ppm fluoride and 200 μM DCCD. After incubation at 37°C in an anaerobic chamber for 24 h, bacterial growth was spectrophotometrically measured with a microtiter plate reader at an absorbance of 600 nm. In the control groups, UA159 and UA159-T were cultivated without DCCD. The OD_600_ readings from medium only wells were subtracted to account for background readings. The experiment was independently repeated four times with two replicates per group (*N* = 8 per group). IBM SPSS Statistics 24 was employed for statistical analysis using independent two-sample *t*-test (two-tailed test) to compared groups. A *P*-value of 0.05 was considered significant. In addition, to further study the effect of gradient-concentration fluoride treatment with 200 μM DCCD on *S. mutans* growth, the same MIC procedure was applied for strains (UA159/FR1000/UA159-T) with 3/4-fold serial dilutions of NaF. In the control groups, strains (UA159/FR1000/UA159-T) were cultivated without DCCD at 37°C in an anaerobic chamber for 24 h. The OD_600_ readings from medium-only wells were subtracted to account for background readings. The experiment was independently repeated three times with four replicates per group (*N* = 12 per group and per fluoride concentration).

### Measurement of Relative Intracellular Fluoride Concentration

The relative intracellular fluoride concentration was measured as previously described (Drescher and Suttie, 1972; Quissell and Suttie, 1972; Li et al., 2013) with some modifications. Briefly, strains (UA159/FR600/FR1000/UA159-T) were grown in TSB until exponential phase (OD_600_ = 0.6). A 24-well culture plate (Corning-Costar, NY, United States) containing 1 mL TSB supplemented with 1/2-fold serial dilutions of non-radioactive NaF and ^18^F (provided by Laboratory of Nuclear Medicine of West China Hospital) was prepared. The concentration of NaF was 8,000 ppm in column 1 and 250 ppm in column 6. The activity of ^18^F in column 1 was 320 μCi and that in column 6 was 10 μCi. 1 mL bacterial suspension was added to each well. Thus, the concentration of NaF in column 1 became 4,000 ppm and in column 6 became 125 ppm. The activity of ^18^F was not altered. After incubation at 37°C in an anaerobic chamber for 1 h, the media were removed, and the cells were washed four times through vacuum filtration. Any fluoride not absorbed by the cells was washed away. Cells were then resuspended in 1 mL PBS, and the radiation intensity was measured by scintillation counting using a ^18^F measurement protocol. Each sample was counted for 1 min, and the decay of ^18^F during counting was accounted for by using the half-life of 109 min. Hundred microliter of a 1:1,000 dilution of the 320 μ Ci ^18^F standard stock (for contrast) was counted alongside the samples (experimental groups: UA159/FR600/FR1000/UA159-T) to determine the specific activity. At the same time, OD_600_ reading of each well, which represents relative number of cells, was measured. The OD_600_ readings from medium only wells were subtracted to account for background readings. The relative concentration of NaF in each cell was determined by dividing the counts by the OD_600_ readings. The experiment was independently repeated three times with two replicates per group (*N* = 6 per group and per fluoride concentration). IBM SPSS Statistics 24 was employed for statistical analysis using One-way ANOVA to compared groups. Dunnett’s T3 was used for multiple comparisons after ANOVA (heterogeneity of variance). A *P*-value of 0.05 was considered significant.

### Rat Caries Models

The *in vivo* effect of *S. mutans* on SPF Wistar rats was assessed using methods previously described (Galvao et al., 2017; Garcia et al., 2017). Twelve 17-days-old male pups free of *S. mutans* were provided by Chengdu Dossy Experimental Animals Co., Ltd. (Chengdu China) and acclimatized for 3 days in our SPF Animal Center. They were randomly divided into two groups with six rats in each group. All animals were provided sterile drinking water containing 0.1% ampicillin, 0.1% chloramphenicol, 0.1% carbenicillin (J&K Chemical, Beijing, China) for 3 days to suppress endogenous flora before the infection of *S. mutans.* Saliva (obtained by sterile cotton swabs) of each rat was plated on BHI to determine the total cultivable microorganisms and to check whether endogenous flora was effectively suppressed. The rats were orally infected with *S. mutans* UA159 and UA159-T using sterile injection syringes containing mid-exponential bacterial cultures of 10^7^ CFU/mL and 0.2 mL per rat for successive days until the oral infection was confirmed 1 week later by PCR (saliva samples obtained by sterile cotton swab, primer atpH-seqFor and atpH-seqRev) and sequencing. See Supplementary Figure 2 for the detailed comparison results of PCR product sequences. During this period, rats were given highly cariogenic Diet 2000# (Trophic Animal Feed High-Tech Co., Ltd., China) and 5% sucrose water *ad libitum*. The oral-infection experiments were conducted by one group member and the following fluoride-treatment experiments and data analysis work were conducted by another group member without knowing the group label. For treatment experiments, rat molars were flushed with fluoride solution (1,000 ppm) for 1 min daily, using 5-mL-sterile injection syringes. Rats were weighed weekly and killed at 4 weeks with CO_2_ asphyxiation. Their maxillas and mandibles were dissected, cleaned and suspended in 0.4% urea amine solution in dark for 12 h for dyeing. All operations were performed in random order to minimize potential confounders.

Caries severities of smooth-surface and sulcal were observed under stereomicroscopy and scored using Keyes method (Keyes, 1958). In the Keyes scoring method, E means the depth of penetration that restricted to enamel only; slight dentinal penetration (Ds) describes penetration overlying enamel and up to one-fourth of the dentin between the enamel and pulp chamber; dentinal penetration between one-fourth and three-fourths is classified as moderate (Dm) while penetration beyond three-fourths is designated as extensive (Dx). Besides cavity depth, cavity extension is another important factor for calculating Keyes’ scores. The buccal, lingual, sulcal and proximal surfaces of rat molars were divided into standard units (Table 1 and Supplementary Figure 3). For each lesion severity level (E,Ds,Dm,Dx), lesion extension units were calculated, respectively, in smooth surfaces (Supplementary Figure 3A) and sulcal surfaces (Supplementary Figure 3B). Linear extent of lesions was judged by eye. And where necessary, lesions were probed to allow estimation. Only whole numbers were used. Group differences were compared by independent two-sample *t*-test (two-tailed test, heterogeneity of variance) using the IBM SPSS Statistics 24. A *P*-value of 0.05 was considered significant. The study conforms to the ARRIVE Guidelines.

**TABLE 1:** The standard units of buccal, lingual, sulcal and proximal surfaces of rat molars.

Lesion types	Mandibular			Maxillary		
		
	1st	2nd	3rd	1st	2nd	3rd
Buccal	6	4	4	6	4	3
Lingual	6	4	4	6	4	3
Sulcal	7	5	2	5	3	2
Proximal	1	2	1	1	2	1

## Results

### High Fluoride Concentrations Caused Fluoride Tolerance in *Streptococcus mutans* Through Point Mutations

**FIGURE 1:**
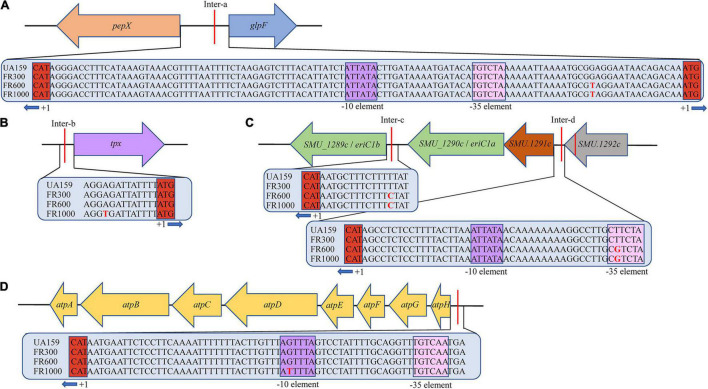
Organization of gene clusters of *S. mutans* and their relation to intergenic SNPs. **(A)** Intergenic SNP upstream of *pepX* and *glpF* gene in FR600/FR1000. **(B)** Intergenic SNP upstream of *tpx* gene in FR1000. The -10A→T promoter mutation occurred in FR1000. **(C)** Intergenic SNP *eriC1a* (*SMU_1290c*) and *eriC1b* (*SMU_1289c*) in FR600 and FR1000. The -13T→C promoter mutation occurred in FR600 and FR1000. Intergenic SNPs of *SMU_1292c* and *SMU_1291c* in FR600 and FR1000. The -44T→G promoter mutation occurred in FR600 and FR1000. **(D)** Intergenic SNPs upstream of *atpHGFEDCBA* in FR1000. The -36G→T promoter mutation occurred in FR600 and FR1000. Sequences of intergenic regions were given in the blue bar. Red letter indicates SNPs; Purple box indicates putative -10 element (Pribnow box, share conserved sequences TATAAT/ATTATA); Pink box indicates putative -35 element (share conserved sequences TTGACA/TGTCAA); Red box indicates translation start site (ATG/CAT) of the operon.

*S. mutans* fluoride-resistant strains thriving in 300–1,000 ppm fluoride were isolated and named FR300 (*SRR13846724*), FR600 (*SRR13846723*), and FR1000 (*SRR13846722*). Though whole-genome sequencing, three types of variant analyses, including structural variations, insertion-deletion variant (InDel) calling and SNP calling, were carried out on the scaffolds of all strains. The resistant strains showed high homology and no genome rearrangement was observed. InDels in different coding regions in the 3 fluoride-resistant strains were compared with UA159 as listed in Supplementary Table 1. The results on SNP analysis, including 4 stopgain single nucleotide variants (SNVs) in 3 Open Reading Frames (ORFs), 1 stoploss SNV, 2 synonymous coding substitutions and 2 non-synonymous coding substitutions, are shown in Supplementary Table 2. The organization of gene clusters and their relations to the intergenic SNPs are shown in Figure 1. Most prokaryotic promoters share conserved sequences at the putative −10 element (TATAAT) and the putative −35 element (TTGACA). These two sequences form RNA polymerase binding site (Browning and Busby, 2004), and mutations at this region may alter RNA polymerase affinity. Notably, one SNP in FR600 and FR1000 occurred in the −35element of the *SMU_1291c* gene and one SNP in FR1000 occurred in the −10 element of the *atpH (SMU_1534)* gene. Interestingly, the *tpx (SMU_924)* SNP (−10A→T) and the *atpH* SNP (−36G→T) occurred only in FR1000, which was resistant to high fluoride concentrations, but not in FR300 or FR600. And the *atpHGFEDCBA* operon codes for a F_0_F_1_-ATPase/ATP synthase, which is a proton pump that maintains intracellular pH of bacteria. This highly suggests that F_0_F_1_-ATPase, whose mutation has not been found even in the renowned fluoride resistant strain UA159FR, could be an important regulation site of bacterial fluoride resistance.

### A Novel Point Mutation in F_0_F_1_-ATPase Promoter Up-Regulated the Expression of *atpH* and Fluoride Resistance

**FIGURE 2:**
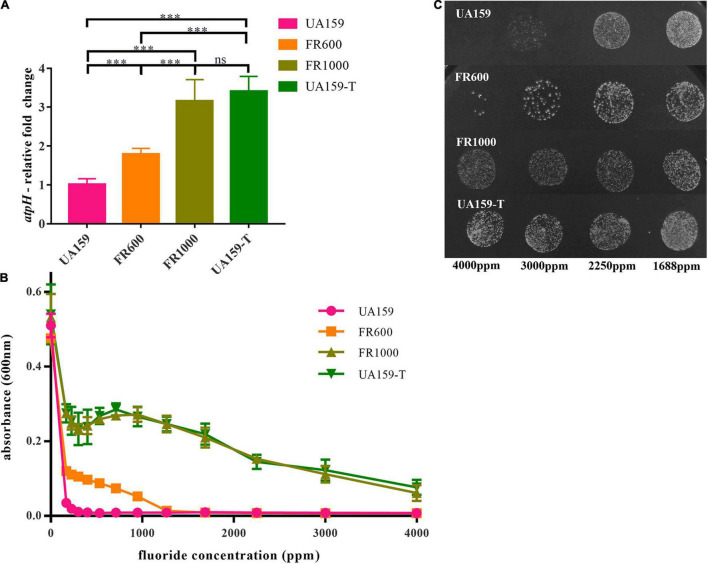
−36G→T point mutation in UA159-T caused up-regulated *atpH* gene expression and fluoride resistance. **(A)** Quantitative real-time PCR of *atpH*: One -36G→T point mutation in F_0_F_1_-ATPase promoter significantly up-regulated *atpH* gene expression. Overall expression compared to UA159 was presented as average fold change ± SD. The significance level (α) was set at 0.05 (One-way ANOVA, Dunnett’s T3, IBeleM SPSS Statistics 24). *** indicates *P* < 0.001. ns indicates no significant difference. *N* = 12 per group. **(B)** Effects of gradient-concentration fluoride treatment on *S. mutans* growth: One -36G→T point mutation in F_0_F_1_-ATPase promoter significantly increased fluoride resistance. 1:100 diluted overnight suspension of strains was incubated in 96-well culture plates with different concentrations of fluoride (3/4-fold serial) for 24 h. *N* = 12 per group and per fluoride concentration. **(C)** Effects of gradient-concentration fluoride treatment on Colony-Forming Units of *S. mutans*: FR1000 and UA159-T showed higher fluoride tolerance than UA159. Five microliter suspension of the UA159, FR1000, UA159-T from the 96-well culture plate of MIC assays (treated with 3/4-fold serial dilutions of fluoride for 24 h) was incubated on TSA for 2 days. Images were taken by a microscopy.

To verify whether F_0_F_1_-ATPase is associated with fluoride resistance of *S. mutans*. A F_0_F_1_-ATPase promoter point mutation (UA159-T) was established from the reference strain UA159. Hereafter, *atpH* gene expression of FR600, FR1000, and UA159-T were compared with the parent strain by quantitative real-time PCR (qRT-PCR) using *gyrA* as an internal control. The *atpH* expression of FR600 was 1.79-fold higher (*P* < 0.001), of FR1000 was 3.15-fold higher (*P* < 0.001) and of UA159-T was 3.40-fold higher (*P* < 0.001) than UA159 (Figure 2A). There were no significant differences between the *atpH* expression of FR1000 and UA159-T (*P* = 0.737).

The MIC to fluoride of UA159 was 200 ppm; of FR600 was 1,000 ppm; of FR1000 and UA159-T was 4,000 ppm (Figure 2B). Apparently, −36G→T mutation in promoter increased F_0_F_1_-ATPase expression, resulting to the increase in fluoride resistance in *S. mutans.* 5 μL suspension of UA159, FR1000, and UA159-T from the 96-well culture plate of MIC assays, was grown anaerobically on TSA agar for 2 days, respectively. Stereomicroscopy images showing the effect of gradient-concentration fluoride treatment on Colony-Forming Units (CFUs) were shown in Figure 2C. After 2 days’ incubation in the fluoride free TSA agar, many colonies were seen in cases of FR1000 and UA159-T, which indicates that for these strains, high-fluoride treatment only have antibacterial effect instead of bactericidal effect. In contrast, almost none colonies were seen in the case of parent strain UA159 after the 4,000 ppm fluoride treatment. These findings indicate fluoride resistance of *S. mutans* can be attributed to the one-point mutation at the promoter region of the *atpHGFEDCBA* operon.

### Suppression of the F_0_F_1_-ATPase Activity by Dicyclohexylcarbodiimide Reduced Fluoride Resistance Induced by the Promoter Mutation

**FIGURE 3:**
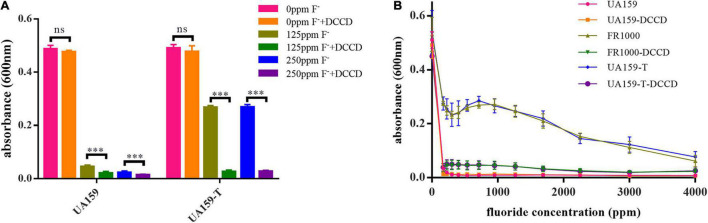
Effects of Dicyclohexylcarbodiimide (DCCD), a specific F_0_F_1_-ATPase inhibitor, on fluoride resistance of *S. mutans*. **(A)** Fluoride inhibitory assays with 200 μM DCCD: suppression of the F_0_F_1_-ATPases by DCCD increased fluoride susceptibility of the fluoride-resistant strains in fluoride environment. The significance level (α) was set at 0.05 (independent two-sample *t*-test, two-tailed test, IBM SPSS Statistics 24). *** indicates *P* < 0.001. ns indicates no significant difference. *N* = 8 per group. **(B)** Effects of gradient-concentration fluoride treatment with 200 μM DCCD on *S. mutans* growth (*N* = 12 per group and per fluoride concentration): 200 μM DCCD suppressed F_0_F_1_-ATPases, increased fluoride susceptibility of the fluoride-resistant strains, and reversed the resistance effects induced by F_0_F_1_-ATPase over-expression.

To further validate the relationship between the increasing of F_0_F_1_-ATPase expression and fluoride resistance, the suppression assay was performed. DCCD, a F_0_F_1_-ATPase specific inhibitor, halts proton translocation across the cell membrane (Belli et al., 1995). In the suppression assay, 200 μM DCCD showed no significant cytotoxic effects in a fluoride-free environment (Figure 3A). There were no significant differences between the fluoride-free groups cultivated with and without DCCD (*P* = 0.370 in the UA159 group; *P* = 0.910 in the UA159-T group). However, 200 μM DCCD suppressed UA159 and UA159-T growth in 125 and 250 ppm fluoride environment (*P* < 0.001). Interestingly, 200 μM DCCD reduced fluoride resistance in both fluoride sensitive strain UA159 and fluoride resistant strain UA159-T (Figure 3A). Addition of 200 μM DCCD reduced the MIC values of FR1000 and UA159-T to the level of UA159 (Figure 3B). Thus, the positive effects that over-expression of F_0_F_1_-ATPase brought about could be offset by suppression of F_0_F_1_-ATPase.

### Fluoride Resistant Strains Tolerated Higher Intracellular Fluoride Concentrations

**FIGURE 4:**
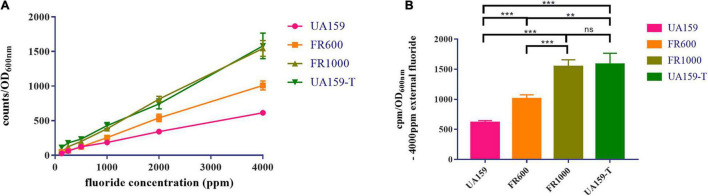
Measurement of relative intracellular fluoride concentration. **(A)** The relative intracellular fluoride concentration increased alongside the external fluoride concentration. **(B)** With the existence of 4,000 ppm external fluoride, relative intracellular fluoride concentrations of fluoride resistant strains (FR1000 and UA159-T) were higher than UA159. The significance level (α) was set at 0.05 (One-way ANOVA, Dunnett’s T3, IBM SPSS Statistics 24). *N* = 6 per group. ** indicates *P* < 0.01. *** indicates *P* < 0.001. ns indicates no significant difference.

Relative intracellular fluoride concentration was measured by radioactive ^18^F and results were shown in Figure 4. In all strains, the relative intracellular fluoride concentration increased with the increase of external fluoride concentration. Strains that are resistant to higher fluoride concentrations had accordingly higher intracellular fluoride concentrations (Figure 4A). With addition of 4,000 ppm external fluoride, intracellular fluoride levels in fluoride resistant strains were higher compared to UA159 (Figure 4B, *P* < 0.001 in FR600/FR1000/UA159-T group when compared to UA159). There was no significant difference in intracellular fluoride concentrations of FR1000 and UA159-T (*P* = 0.998). In conclusion, fluoride resistant strains could tolerate higher intracellular fluoride concentrations than fluoride sensitive strains.

### −36G→T Mutation in F_0_F_1_-ATPases Promoter Enhanced the Cariogenicity of *Streptococcus mutans* Under Fluoride Exposure

**FIGURE 5:**
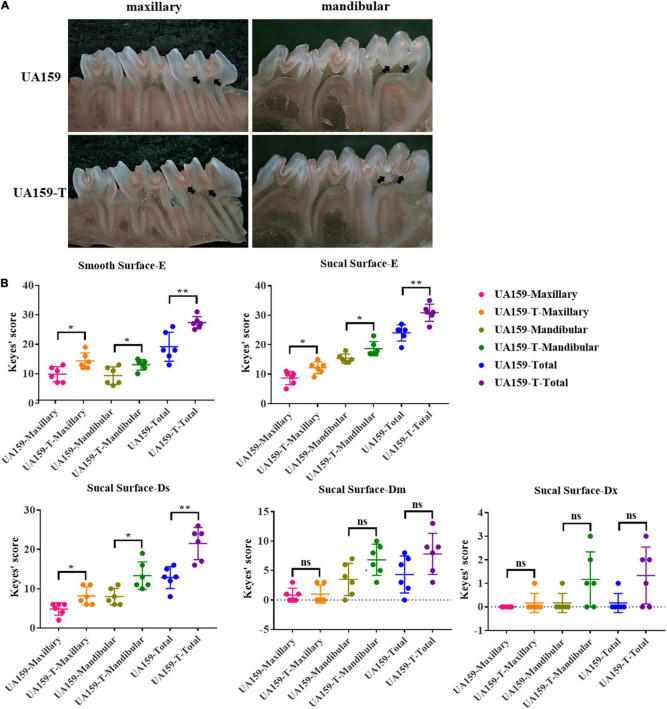
Dental caries conditions and Keyes’ scores of rat caries models. **(A)** Stereomicroscopy images of dental caries on maxillary and mandibular bones of rats infected with UA159 and UA159-T. Black arrows point out different severity in the same site of the two groups. UA159-T strains led to severer dental caries than UA159. **(B)** Keyes’ scores of smooth surfaces and sulcal surfaces of two group (UA159 and UA159-T) and *N* = 6 per group: UA159-T strains achieved higher Keyes’ scores and led to severer dental caries than UA159. The significance level (α) was set at 0.05 (independent two-sample *t*-test, two-tailed test, IBM SPSS Statistics 24). * indicates *P* < 0.05. ** indicates *P* < 0.01. ns indicates no significant difference.

To examine the cariogenicity of the fluoride resistant strains, we established rat caries models inoculating these strains. Different effects of UA159 and UA159-T on maxillary and mandibular teeth were illustrated in Figure 5A. Black arrows showed different severity in the same site of the two groups. As shown in the image, UA159-T caused more severe dental caries than UA159.

Keyes’ scores of dental caries on smooth surface were calculated, analyzed and shown in Figure 5B and Supplementary Tables 5, 6. Since caries on the smooth surfaces were not severe, the lesion extension was restricted to enamel regions only. Smooth surface scores (E) showed significant differences between UA159 and UA159-T in maxilla (*P* = 0.015), mandibula (*P* = 0.033) and in total (*P* = 0.008). Keyes’ scores of dental caries on sulcal surface were calculated, analyzed and presented as graphs (Figure 5B). Maxillary, mandibular and accumulated sulcal enamel lesions (E) and slight dentinal lesions (Ds) scores for UA159-T group were significantly higher compared with UA159 group scores (*P* = 0.018 in maxilla/E; *P* = 0.020 in mandibula/E; *P* = 0.002 in total/E; *P* = 0.018 in maxilla/Ds; *P* = 0.012 in mandibula/Ds; *P* = 0.002 in total/Ds). Moderate dentinal (Dm) scores for UA159-T group, though seemed to be higher, were not significantly different from UA159 group scores (*P* = 0.838 in maxilla/Dm; *P* = 0.057 in mandibula/Dm; *P* = 0.098 in total). As for extensive lesions (Dx), as only few animals had Dx lesions probably because animals were reared over a short period. Most animals in both groups had a Dx score of zero. In UA159-T group, a few animals showed Dx scores up to 3. However, Dx scores showed no significance difference in maxilla (*P* = 0.363), mandibula (*P* = 0.094) and in total (*P* = 0.066) between the two groups. From these findings, we conclude that fluoride resistant strains developed by over-expressed F_0_F_1_-ATPase caused more severe dental caries compared to the reference strain.

## Discussion

Fluoride is an abundant element in environment and is added as an anti-caries agent to a variety of oral care products (Anusavice et al., 2016). Fluoride is absorbed by bacterial cells in the form of HF, which dissociates in the cell into F^–^ and H^+^. Intracellular F^–^ and H^+^ directly or indirectly affects physiological processes and enzymatic activities in the cell, such as F_0_F_1_-ATPase, Enolase, fluoride exporters, pyruvate kinase, pyrophosphatase, urease and the arginine deiminase system (ADS) (Rolla and Melsen, 1975; Bender et al., 1985; Meurman, 1988; Marquis, 1990; Belli et al., 1995; Van Loveren, 2001; Hamilton and Ellwood, 1978). Many microorganisms have developed fluoride resistance (Streckfuss et al., 1980; Mitsuhata et al., 2014; Liao et al., 2015) due to extensive usage of fluoride in oral hygiene products. As a result, these products are not expectedly effective in treatment of dental caries. Stable fluoride resistant strains were isolated and used to study the mechanism of the acquired resistance, which is still unclear.

In this study, the effects of fluoride treatment on *S. mutans* were studied through comparison of functional genomics. Fluoride treatment on *S. mutans* strain UA159 resulted to 4 fluoride-resistant strains (FR300, FR600, FR1000, and UA159-T) and a site-directed mutagenesis (-36G→T) in the F_0_F_1_-ATPases promoter region in UA159-T. Two SNPs in FR300, 8 SNPs in FR600 and 9 SNPs in FR1000 were identified through the Illumina PE250 sequencing platform and compared with UA159 (Supplementary Table 2).

Notably, a non-synonymous SNP was identified in FR600 and FR1000 in *pykF (SMU_1190)* gene, which encodes pyruvate kinase, an important glycolytic enzyme. Enolase catalyzes the conversion of 2-phosphoglycerate to phosphoenolpyruvate (PEP), while pyruvate kinase converts PEP to pyruvate during glycolysis. Fluoride competitively inhibits enolase enzyme (Guha-Chowdhury et al., 1997). This inhibition is observed both in purified enolase and enolase from permeabilized cells (Curran et al., 1994; Guha-Chowdhury et al., 1997; van Loveren et al., 2008). In addition, enolase is indirectly inhibited by low cytoplasm pH levels caused by H^+^ accumulation (Belli et al., 1995; Marquis et al., 2003). Moreover, enolase catalyzes the production of PEP for glucose uptake through the PEP dependent phosphotransferase system (PTS). Thus, enolase inhibition results in reduction of sugar uptake as well as glycolysis (Sutton et al., 1987). Previous studies (Marquis et al., 2003) reported that inhibition of pyruvate kinase is unlikely to occur in bacterial cells because the inhibitory fluoride concentration for this enzyme was approximately 10–100 times higher than that required for enolase inhibition. Liao et al. (2015, 2018) reports two different SNPs from ours in *S. mutans pykF* gene using whole-genome shotgun sequencing and genome comparisons between C180-2 and C180-2FR. However, qRT-PCR analysis shows similar pyruvate kinase expression levels in C180-2 and C180-2FR. Thus, the role of pyruvate kinase in bacterial fluoride resistance needs further explore.

Genes controlled by promoters in the mutated intergenic regions were identified (Supplementary Table 2 and Figure 1). Firstly, *glpF (SMU_396)* and *pepX (SMU_395)* genes located downstream of the mutated intergenic region were identified (Inter-a, Figure 1). *pepX* encodes Xaa-Pro dipeptidyl-peptidase (Q8DVS2), which cleaves N-terminal dipeptides sequentially from polypeptides with unsubstituted N-termini provided that the penultimate residue is proline. *glpF* encodes a putative glycerol uptake facilitator protein, a membrane channel that selectively transports water, small neutral molecules and ions (Fu et al., 2002). In *Escherichia coli*, which uses glycerol as a carbon source, *glpF* mediates glycolysis and lipid biogenesis (Heller et al., 1980). In *Lactococcus lactis*, it mediates the growth of bacterial cells in the presence of glycerol (Froger et al., 2001). In this study, we identified the same SNP in the intergenic region of *pepX* and *glpF* genes as the previously reported SNP by Liao et al. (2015) in *S. mutans* C180-2 and C180-2FR. In their study, Liao et al. (2015) reported lower expression levels of the *glpF* in C180-2FR than C180-2 in early exponential phase. The lower expression levels may be attributed to slower growth rate of the fluoride-resistant strain. In conclusion, *glpF* plays a role in bacterial cell growth but not in fluoride resistance.

A mutation in an intergenic region (Supplementary Table 2 and Inter-b, Figure 1) was located upstream of *tpx* gene, which encodes thiol peroxidase. Thiol-specific peroxidase catalyzes the reduction of hydrogen peroxide and organic hydroperoxides to water and alcohols. It protects cells against oxidative stress by breaking down peroxides (Baker and Poole, 2003; Cha et al., 2004). Interestingly, the -10A→T promoter mutation only occurred in FR1000 but not in FR300 or FR600. Thus, there is a high possibility that this mutation may be implicated in fluoride resistance and further exploration is needed.

Mutations were observed between *SMU_1289c* and *SMU_1290c* genes (Supplementary Table 2 and Inter-c, Figure 1) and between *SMU_1291c* and *SMU_1292c* genes (Supplementary Table 2 and Inter-d, Figure 1). These 3 genes, *SMU_1291c*, *SMU_1290c/eriC1a* and *SMU_1289c/eriC1a* occur in proximity and they encode putative chorismate mutase, putative permease and chloride channel, respectively. Breaker (2012)x and Baker et al. (2012) reported that *crcB* (fungal *FEX* homologs) and EriC*^F^* (ClC-type ion channel protein) gene families play similar biochemical roles. These gene families encode fluoride exporters (Rapp et al., 2006; Baker et al., 2012) and are implicated in fluoride resistance of microorganisms. These genes are divided into three groups: group I, consisting of *eriC1*; group II, *eriC1* and *eriC2*; and group III, *eriC2*, *crcB1*, and *crcB2*. A previous study reported that these genes were highly selective fluoride channels that discriminate against Cl^–^ by a factor of > 10,000-fold (Stockbridge et al., 2013). Unlike other CLC transporters, which employ two-to-one stoichiometry, the fluoride exporter exchanges F^–^ with H^+^ with one-to-one stoichiometry (Picollo et al., 2012; Stockbridge et al., 2012). *eriC1* in group I is from *S. mutans* and has two *eriC^F^* genes in tandem with the same orientation, namely *eriC1a* and *eriC1b* (Men et al., 2016). The two genes encode proteins with 58% amino acid identity (Liao et al., 2015). Ying Liao reported the same −44T→G SNP in C180-2FR (Liao et al., 2015) as our study (Supplementary Table 2 and Inter-d, Figure 1), and a different -47T→A SNP (Liao et al., 2018) in UA159FR. Expression levels of the three genes was higher in both C180-2FR and UA159FR compared with C180-2 and UA159. A -44T→G mutation in the promoter of strain C180-2 constitutively upregulated *eriC1a* and *eriC1b* expression and conferred fluoride resistance on fluoride sensitive *S. mutans* strain (Liao et al., 2016). Two gene knockout studies demonstrated that *eriC1a* and *eriC1b* genes were implicated in fluoride resistance (Men et al., 2016; Murata and Hanada, 2016). *S. mutans* strains with *eriC^F^* genes knocked out were more susceptible to fluoride in both studies. However, one study showed knocking out either *eriC1a* or *eriC1b* gene can increase fluoride susceptibility (Murata and Hanada, 2016) while another author reported that only *eriC1b* gene was implicated in fluoride resistance (Men et al., 2016). In conclusion, *eriC1a* and *eriC1b* encode H^+^-coupled fluoride antiporters EriC*^F^*, which extrudes F^–^ from cell. High expression levels of these two genes protect *S. mutans* fluoride resistant strains from high fluoride concentrations.

Furthermore, a mutated intergenic region (Supplementary Table 2 and Inter-d, Figure 1) located upstream of *atpHGFEDCBA*, which separately encodes ATP synthase subunit proteins, was observed. ATP synthase, also known as F_0_F_1_-ATPase, is a protein consisting of two domains: F_0_ (cytoplasmic) and F_1_ (membrane bound). F_0_F_1_-ATPase hydrolyzes or synthesizes ATP while transporting protons through the F_0_ pore in the membrane (Bender et al., 1986). Therefore, F_0_F_1_-ATPase plays an important role in regulating intracellular pH through proton transporting and is closely related to the acid tolerance of bacteria (Bender et al., 1986). Differences in the pH optima of F_0_F_1_-ATPase appears to be the main reason why *S. mutans* is more tolerant of low pH values and hence pathogenic. Previous studies have shown that the pH optima of F_0_F_1_-ATPase of *S. mutans* was approximately 6.0 while that of *Streptococcus sanguis* was approximately 7.0 (Sturr and Marquis, 1992). Thus, the S. *mutans* enzyme is well positioned to continue pumping at pH values below levels at which *S. sanguis* can survive. The expression of F_0_F_1_-ATPase gene of *S. mutans* is regulated by pH and will be up-regulated in acidic environment than in neutral environment (Kuhnert et al., 2004). As for fluoride, previous studies found that it can inhibit F_0_F_1_-ATPases directly by F^–^ and indirectly by cytoplasm acidification through HF influx (Liao et al., 2017). Inhibiting proton transporting of F_0_F_1_-ATPases (dissipating pH_*i*_ gradients) is one important mechanism of fluoride to prevent dental caries (Dashper and Reynolds, 1992). However, the role of F_0_F_1_-ATPase in fluoride resistance has never been reported. Researchers speculated that, fluoride-resistant *S. mutans* are able to up-regulate synthesis of its F_0_F_1_-ATPase (Miwa et al., 1997; Belli and Marquis, 2010; Hamilton and Buckley, 2010). In order to better understand the molecular mechanisms by which *S. mutans* up-regulates its F_0_F_1_-ATPase, Smith et al. (1996) had previously undertaken the characterization of the *S. mutans atpHGFEDCBA* operon, including its cloning and nucleotide sequence determination. The deduced amino acid sequences for the eight structural genes of the *S. mutans atpHGFEDCBA* operon showed that this enzyme is homologous to the well-characterized *Escherichia coli* ATPase as well as those of other bacteria (Smith et al., 1996). Interestingly, the *S. mutans* operon did not contain an *atpl* gene equivalent upstream of the structural genes but rather contained an intergenic region of 239 bp (Smith et al., 1996). Kuhnert and Quivey (2003) reported that the genetic organization of the operons has been maintained in the oral streptococci and speculated that the large intergenic regions seen upstream of the *S. mutans atpHGFEDCBA* operons may be involved in the regulation of the operon. Primer extension analysis were used to examine the transcriptional start site for the operon. The results of identified a guanine at position −28 bp relative to the initial methionine for the *atpH* gene. Once the start site was identified, sequence analysis of the region immediately upstream suggested a putative Pribnow box (−10 element) with a sequence of TAAACT, which was similar to the *E. coli* consensus sequence of TATAAT (66% homogeneity). A potential −35 element sequence was also identified that was completely identical to the canonical *E. coli* −35 region of TTGACA (Kuhnert and Quivey, 2003). However, in previous studies on fluoride-resistant strains, no mutation was found in the *atpHGFEDCBA* operon.

In our study, a novel −36G→T promoter mutation was observed in the putative Pribnow box/-10 element (Supplementary Table 2 and Figure 1). Pribnow box is an important RNA polymerase binding site (Browning and Busby, 2004), and mutations at this region may alter RNA polymerase affinity. This novel mutation only occurred in FR1000 but not in UA159, FR300 or FR600, highly suggesting that F_0_F_1_-ATPase could be an important regulation site for bacteria to resist high fluoride concentrations. Quantitative real-time PCR assays showed that *atpH* expression levels were higher in FR600, FR1000 and UA159-T (*P* < 0.001) than in UA159 (qRT-PCR, Figure 2). Interestingly, −36G→T point mutation in the promoter increased of F_0_F_1_-ATPase expression levels (qRT-PCR, Figure 2) and conferred fluoride resistance on the fluoride sensitive *S. mutans* strain (MIC assays, Figure 2). However, the addition of 200 μM DCCD, a specific inhibitor of F_0_F_1_-ATPase, reduced the MIC values of FR1000 and UA159-T to the same amount of UA159 (Figure 3). This implies that 200 μM DCCD reverses the resistance effects induced by F_0_F_1_-ATPase over-expression. F_0_F_1_-ATPase high expression levels may improve fluoride resistance by extruding proton out of the cell thus increasing cytoplasm pH. To be specific, inhibitory effect of fluoride on bacterial intracellular metabolism is modulated by HF influx. Intracellular F^–^ and H^+^ directly or indirectly affects enzymatic activities and physiological processes in the cell. When cells are exposed to high fluoride environment, fluoride antiporters export F^–^ to extracellular by acting as H^+^-coupled antiporters. However, entry of H^+^ ions lower cytoplasm pH. In this case, high expression of F_0_F_1_-ATPase can extrude surplus protons out of cell and increase cytoplasm pH. Thus, F_0_F_1_-ATPase plays an important role in antagonizing fluoride inhibition effect on *S. mutans* and improving fluoride resistance in *S. mutans.* When proton transporting of F_0_F_1_-ATPases was inhibited by DCCD, surplus protons cannot be pumped out of the cell, causing acidification of the cytoplasm, and further affecting physiological processes of the cell. What is more, the study founded the relative intracellular fluoride concentration of fluoride resistant strains was higher than in UA159, implying that fluoride resistant strains could tolerate higher intracellular fluoride concentrations than fluoride sensitive strains (Figure 4). This finding supported our hypothesis that the increase of fluoride tolerance was caused by increasing H^+^ extrusion and cytoplasm pH. Rat caries models under fluoride exposure showed that UA159-T caused more severe dental caries than UA159 (Figure 5). In summary, tolerance of *S. mutans* to high fluoride concentrations is speculated to be caused by critical genetic changes. The novel SNP in the F_0_F_1_-ATPases promoter can greatly increase fluoride resistance and cariogenicity in *S. mutans.* High correlation between F_0_F_1_-ATPase suppression and fluoride susceptibility makes F_0_F_1_-ATPase a potential suppression target for fluoride resistant strains.

## Data Availability Statement

The datasets presented in this study can be found in online repositories. The names of the repository/repositories and accession number(s) can be found below: NCBI SRA BioProject, accession no: PRJNA706102.

## Ethics Statement

All animal experiments were performed in strict accordance with guidelines from the Institutional Animal Care and Use Committee (IACUC). The animal study was reviewed and approved by Ethics Committee of State Key Laboratory of Oral Diseases, Sichuan University, Chengdu, China (Protocol WCHSIRB-D-2019-184).

## Author Contributions

CL, XX, and XP contributed to conception, design, data acquisition, analysis, and interpretation, drafted, and critically revised the manuscript. ZL, CQ, and SY contributed to data acquisition, analysis, and interpretation, and drafted the manuscript. BR, JL, XZ, and HC contributed to design and critically revised the manuscript. All authors gave final approval and agreed to be accountable for all aspects of the work.

## Conflict of Interest

The authors declare that the research was conducted in the absence of any commercial or financial relationships that could be construed as a potential conflict of interest.

## Publisher’s Note

All claims expressed in this article are solely those of the authors and do not necessarily represent those of their affiliated organizations, or those of the publisher, the editors and the reviewers. Any product that may be evaluated in this article, or claim that may be made by its manufacturer, is not guaranteed or endorsed by the publisher.
