# Supplementary material for: F0F1-ATPase Contributes to the Fluoride Tolerance and Cariogenicity of Streptococcus mutans
Source: Front Microbiol. 2022 Jan 31;12:777504. doi: 10.3389/fmicb.2021.777504 (PMC8841791; doi:10.3389/fmicb.2021.777504)
Supplement: Supplementary file 1 [file Data_Sheet_1.docx]

Supplementary Material

# Supplementary Figures and Tables

## Supplementary Figures


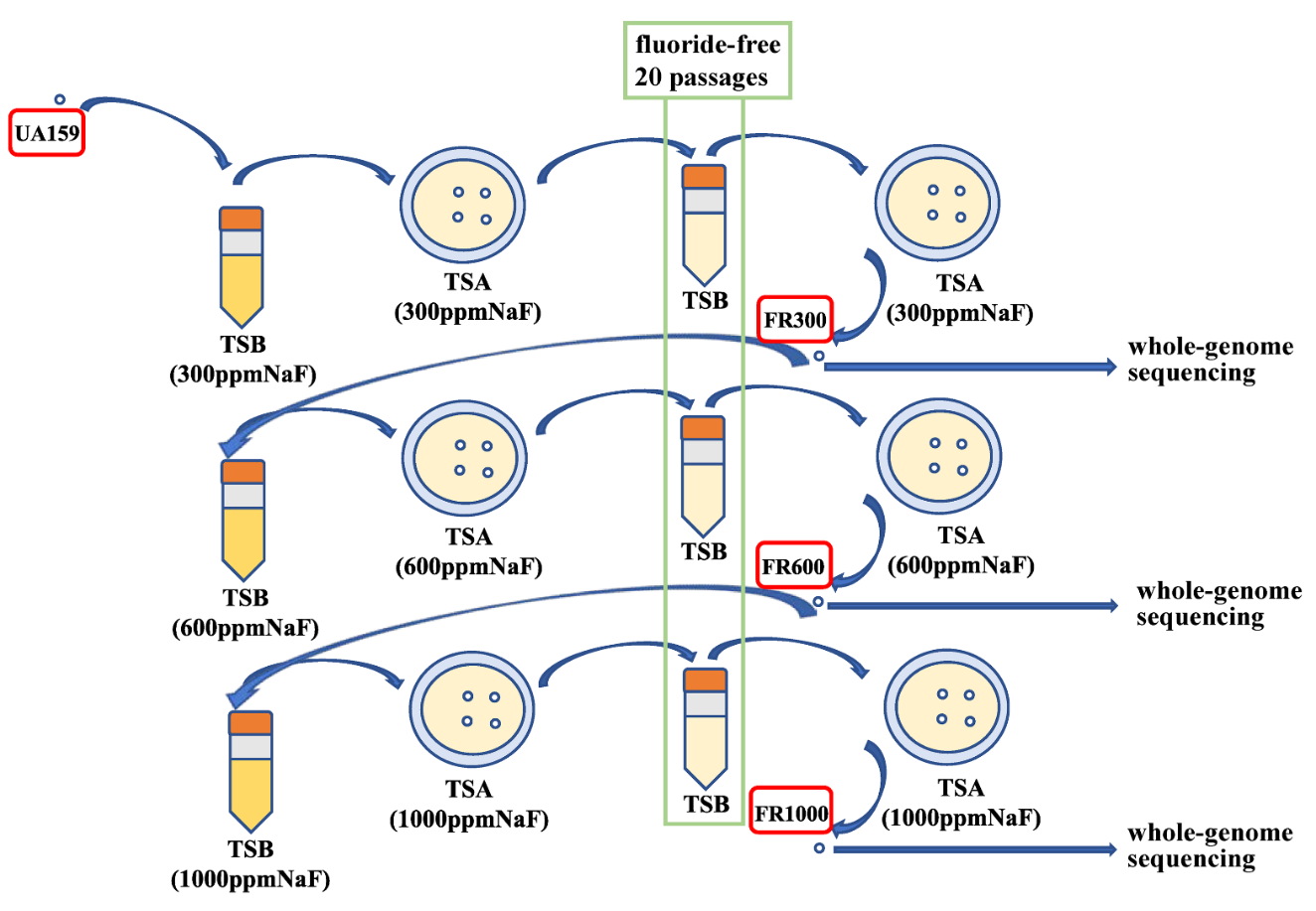


**Fig S1.** Fluoride-resistant strains (FR300, FR600 and FR1000) were obtained through a stepwise procedure in laboratory.


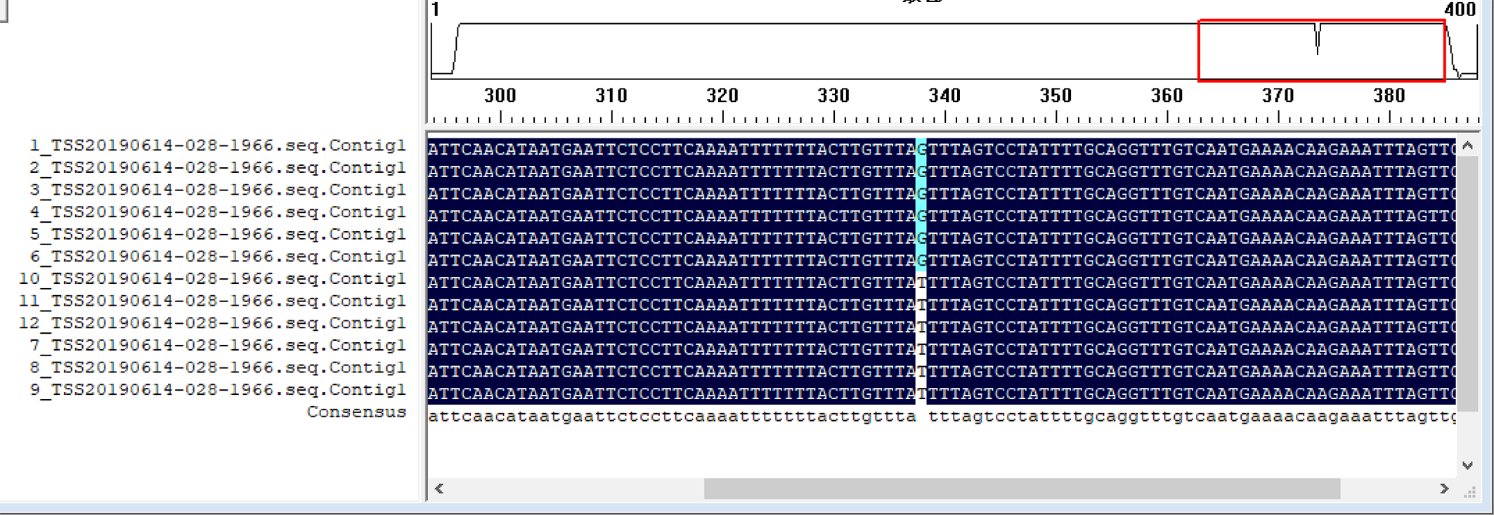


**Fig S2.**Comparison results of PCR product sequences of rats successfully orally infected with *S. mutans* UA159 (sample 1-6) and UA159-T (sample 7-12).


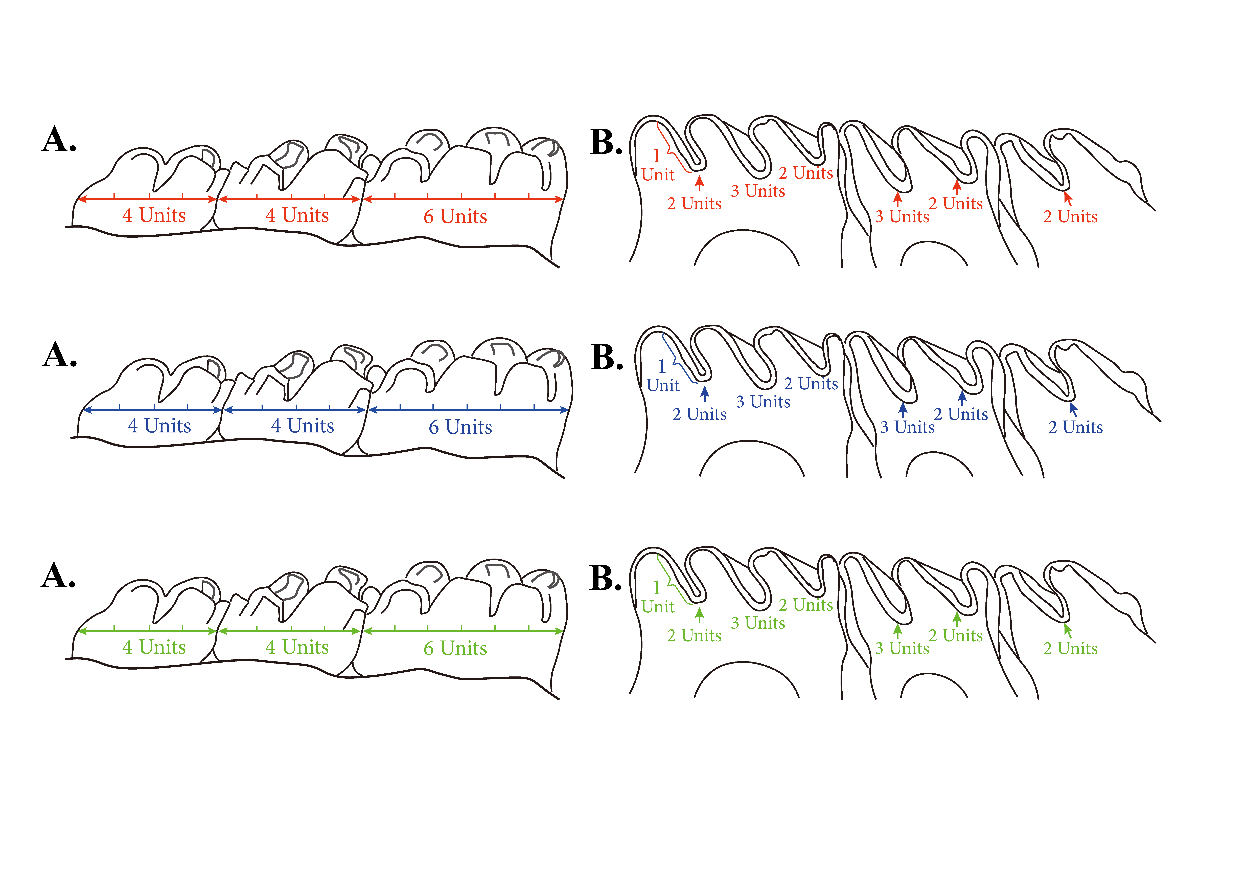


**Fig S3.** Schematic diagram of the standard units of mandibular rat molars. The number of sulci examined in the mandibular first, second, and third molars is 3, 2, and 1, respectively; and in maxillary first, second, and third molars is 2, 1, and 1, respectively. (A). Standard units of smooth surfaces. (B). Standard units of sulcal surfaces.

## SupplementaryTables

**Table S1.** InDels in the genome of *S. mutans* UA159, FR300, FR600 and FR1000.

| UA159 | |  | **FR300** | |  | **FR600** | |  | **FR1000** | |  | **START** | **END** | **InDel type^a^** |
| --- | --- | --- | --- | --- | --- | --- | --- | --- | --- | --- | --- | --- | --- | --- |
| REF | **ALT** |  | **REF** | **ALT** |  | **REF** | **ALT** |  | **REF** | **ALT** |  |  |  |  |
| A | - |  | A | - |  | A | - |  | A | - |  | 552448 | 552448 | D1 |
| - | G |  |  |  |  |  |  |  |  |  |  | 770285 | 770285 | I1 |
|  |  |  |  |  |  |  |  |  | ACAGT | - |  | 848130 | 848134 | D5 |
| AATAAT | - |  | AATAAT | - |  | AATAAT | - |  | AATAAT | - |  | 1892328 | 1892333 | D6 |
| - | T |  | - | T |  |  | - | T | - | T |  | 1932162 | 1932162 | I1 |
| REF: reference base; ALT: alternate base  a: InDel type indicates the number of bases inserted (I) or deleted (D). | | | | | | | | | | | | | | |

**Table S2.** SNPs in the genome of FR300, FR600 and FR1000 compared with *S. mutans* UA159.

| FR300 | |  | **FR600** | |  | **FR1000** | |  | **location** | **Mutation type** | **Gene (in or nearby)** |
| --- | --- | --- | --- | --- | --- | --- | --- | --- | --- | --- | --- |
| REF | **ALT** |  | **REF** | **ALT** |  | **REF** | **ALT** |  |  |  |  |
|  |  |  | G | T |  | G | T |  | 372531 | intergenic^a^ | *glpF*: Putative glycerol uptake facilitator protein  *pepX*: Xaa-Pro dipeptidyl-peptidase |
|  |  |  | C | A |  |  |  |  | 556328 | stopgain | *pbp2b*: Penicillin-binding protein 2b |
|  |  |  | C | T |  | C | T |  | 748799 | stopgain | *SMU_803c*: Putative ABC transporter, ATP-binding protein |
|  |  |  | G | A |  |  |  |  | 807060 | nonsynonymous | *pyrA*: Carbamoyl-phosphate synthase small chain |
|  |  |  |  |  |  | A | T |  | 878761 | intergenic^b^ | *tpx*: Thiol peroxidase |
|  |  |  |  |  |  | G | A |  | 1039062 | synonymous | *opuBc*: Putative choline ABC transporter, osmoprotectant binding protein |
|  |  |  | C | G |  | C | G |  | 1130795 | nonsynonymous | *pykF*: Pyruvate kinase |
|  |  |  | T | C |  | T | C |  | 1216401 | intergenic^c^ | *SMU_1289c*: EriC1b, Putative permease, chloride channel,  *SMU_1290c*: EriC1a, Putative permease, chloride channel |
|  |  |  | T | G |  | T | G |  | 1218000 | intergenic^d^ | *SMU_1291c*: Putative chorismate mutase  *SMU_1292c*: Uncharacterized protein |
| T | A |  |  |  |  |  |  |  | 1218003 | stoploss | *SMU_1292c*: Uncharacterized protein |
|  |  |  |  |  |  | G | T |  | 1459583 | intergenic^e^ | *atpHGFEDCBA*: ATP synthase subunit |
|  |  |  | C | A |  |  |  |  | 1784345 | synonymous | *SMU_1897*: Putative ABC transporter, ATP-binding protein |
|  |  |  |  |  |  | C | A |  | 1856878 | stopgain | *rpoC*: DNA-directed RNA polymerase subunit beta' |
| G | T |  |  |  |  |  |  |  | 1856888 | stopgain | *rpoC*: DNA-directed RNA polymerase subunit beta' |
| REF: reference base; ALT: alternate base  a This intergenic region is located between the *pepX* and *glpF* genes.  b This intergenic region is located upstream of *tpx (SMU_924)* gene.  c This intergenic region is located between the *eriC1a* (*SMU_1290c*) and *eriC1b* (*SMU_1289c*) genes.  d This intergenic region is located between the *SMU_1291c* and *SMU_1292c* genes.  e This intergenic region is located upstream of *atpH* gene. | | | | | | | | | | | |

**Table S3.** Bacterial strains used in this study.

| ***S. mutans* strains** | **Relevant characteristic(s)** | **Source or reference** |
| --- | --- | --- |
| UA159 | Wild type; Erm^s^ | American Type Culture Collection (ATCC) |
| FR300 | Fluoride-resistant strain (300ppm) derived from UA159 | This study |
| FR600 | Fluoride-resistant strain (300ppm) derived from FR300 | This study |
| FR1000 | Fluoride-resistant strain (300ppm) derived from FR600 | This study |
| UA159-T | F-ATPases promoter -36G→T mutant derived from UA159 | This study |

**Table S4.** Primer Sequences used in this study

| **Primer Name** | **Sequence** | **Purpose** |
| --- | --- | --- |
| **atpH-For** | 5’ TGTTGAAGCAAGAAGCACGAAA 3’ | qRT-PCR |
| **atpH-Rev** | 5’ TGCAGCTCGTCAGCCTGAAA 3’ | qRT-PCR |
| **tpx-For** | 5’ GCGGTGCAGCTGGTTTGAAT 3’ | qRT-PCR |
| **tpx-Rev** | 5’ AGGGCAGCGTCATAGTTGGG 3’ | qRT-PCR |
| **gyrA-For** | 5’ ATTGTTGCTCGGGCTCTTCCAG 3’ | qRT-PCR |
| **gyrA-Rev** | 5’ ATGCGGCTTGTCAGGAGTAACC 3’ | qRT-PCR |
| **atpH- pTune** | 5’CAAACCTGCAAAATAGGACTANNNNNAACAAGTAAAAAAATTTTGAAGGA 3’  （N indicates degenerate base, a random combination of A T C G） | Mutation construction |
| **atpH-seqFor** | 5’ AGACATCATCAAGATGGTTA 3’ | Mutation construction |
| **atpH-seqRev** | 5’ AAGTCTAAGAAAACGCAACTA 3’ | Mutation construction |

**Table S5..** Influence of fluoride sensitive/resistant strains on development of dental caries on smooth surface (including buccal, lingualand proximal surfaces)in Wistar rats (Keyes’ score).Since caries on the smooth surfaces were not severe, only Keyes’ scoresfor lesion extension restricted to enamel regions (E) were calculated.

|  |  | Lesion extension（E） | |
| --- | --- | --- | --- |
|  | **Strains** | **UA159** | **UA159-T** |
| Buccal | **Maxillary** | 6.2±1.3 | 8.7±0.8 |
|  | **Mandibular** | 5.3±1.2 | 8.2±1 |
|  | **Total** | 12±2.2 | 17±0.4 |
| Lingual | **Maxillary** | 3.3±1.2 | 5.2±1.2 |
|  | **Mandibular** | 2.7±0.8 | 2.7±0.5 |
|  | **Total** | 6±2 | 7.8±1.6 |
| Proximal | **Maxillary** | 0.3±0.5 | 0.5±1.2 |
|  | **Mandibular** | 1.3±1.5 | 2.2±1.3 |
|  | **Total** | 1.7±1.9 | 2.7±1.8 |
| Smooth Surface | **Maxillary** | 9.8±2.6 | 14±2.7 |
|  | **Mandibular** | 9.3±3 | 13±1.8 |
|  | **Total** | 19±4.9 | 27±2.1 |

**Table S6.**Influence of fluoride sensitive/resistant strains on development of dental caries on sulcal surface in Wistar rats (Keyes’ score).

| Strains |  | | UA159 | | | |  |  |  | | UA159-T | | | |
| --- | --- | --- | --- | --- | --- | --- | --- | --- | --- | --- | --- | --- | --- | --- |
|  | Lesion extension |  | | Lesion severity | | |  |  | Lesion extension |  | | Lesion severity | | |
|  | E |  | | Ds | Dm | Dx |  |  | E |  | | Ds | Dm | Dx |
| Maxillary | 8.7±2.3 |  | | 4.8±1.6 | 0.8±1.2 | 0 |  |  | 12.2±2.0 |  | | 8.2±2.3 | 1.0±1.5 | 0.2±0.4 |
| Mandibular | 15.3±1.5 |  | | 8.0±2.1 | 3.5±2.7 | 0.2±0.4 |  |  | 18.7±2.4 |  | | 13.3±3.5 | 6.8±2.6 | 1.2±1.2 |
| Total | 24±2.8 |  | | 12.8±2.8 | 4.3±3.1 | 0.2±0.4 |  |  | 30.8±2.9 |  | | 21.5±4.1 | 7.8±3.5 | 1.3±1.2 |
